# Modulation of Intestinal Functions Following Mycotoxin Ingestion: Meta-Analysis of Published Experiments in Animals 

**DOI:** 10.3390/toxins5020396

**Published:** 2013-02-21

**Authors:** Bertrand Grenier, Todd J. Applegate

**Affiliations:** 1 Department of Animal Sciences, Purdue University, West Lafayette, IN 47907, USA; E-Mail: bgrenie@purdue.edu; 2 Biomin Research Center, Tulln 3430, Austria

**Keywords:** mycotoxin, gastrointestinal tract, nutrients, gut permeability, mucosal immunity, gut microbiota

## Abstract

Mycotoxins are secondary metabolites of fungi that can cause serious health problems in animals, and may result in severe economic losses. Deleterious effects of these feed contaminants in animals are well documented, ranging from growth impairment, decreased resistance to pathogens, hepato- and nephrotoxicity to death. By contrast, data with regard to their impact on intestinal functions are more limited. However, intestinal cells are the first cells to be exposed to mycotoxins, and often at higher concentrations than other tissues. In addition, mycotoxins specifically target high protein turnover- and activated-cells, which are predominant in gut epithelium. Therefore, intestinal investigations have gained significant interest over the last decade, and some publications have demonstrated that mycotoxins are able to compromise several key functions of the gastrointestinal tract, including decreased surface area available for nutrient absorption, modulation of nutrient transporters, or loss of barrier function. In addition some mycotoxins facilitate persistence of intestinal pathogens and potentiate intestinal inflammation. By contrast, the effect of these fungal metabolites on the intestinal microbiota is largely unknown. This review focuses on mycotoxins which are of concern in terms of occurrence and toxicity, namely: aflatoxins, ochratoxin A and *Fusarium* toxins. Results from nearly 100 published experiments (*in vitro*, *ex vivo* and *in vivo*) were analyzed with a special attention to the doses used.

## Abbreviations

AF: Aflatoxins
APC: Antigen-Presenting Cell
DON: Deoxynivalenol
FB: Fumonisins
FUS: *Fusarium* toxins
GALT: Gut-Associated Lymphoid Tissue
GIT: Gastrointestinal Tract
GLUT2: facilitated glucose transporter
GLUT5: fructose transporter
IEC: Intestinal Epithelial Cell
OTA: Ochratoxin A
PP: Peyer’s Patches
SGLT1: sodium-dependent glucose cotransporter 1
TCT: Trichothecenes
TEER: Transepithelial Electrical Resistance
TJ: Tight Junction
T-2: T-2 toxin
UC: Ussing Chamber
ZEA: Zearalenone

## 1. Introduction

Due to the rapid increase of the world population, animal productivity and feed security are becoming major challenges. The demand in feed supply is considerable, and more than 70% of cereal crops are dedicated to animal production. Therefore, any factor that affects the security of the feed supply is a significant constraint to production. Feed spoilage by fungi is not a new problem, but due to their great adaptability these microorganisms are posing a serious risk to the animal feed industry. Recently, fungi have been designated as a greater threat to animal, plant and ecosystem health than the other taxonomic classes of pathogens [1]. In addition, the affected commodity may become contaminated with toxic secondary fungal metabolites, known as mycotoxins. Mycotoxins are structurally diverse low-molecular weight metabolites produced by various molds belonging chiefly to species of the *Aspergillus*, *Penicillium* and *Fusarium* genera. These toxins inflict loss to farmers and reduce the value of contaminated feeds. For example, in the USA, the economic cost due to three mycotoxins (aflatoxins, fumonisins and deoxynivalenol) is estimated to be USD 900 million per year [2]. Effects in animals following the ingestion of these fungal compounds vary from acute, overt disease with high morbidity and death to chronic, decreased resistance to pathogens and reduced animal productivity [3,4]. However, the major problem associated with animal feed contaminated with mycotoxins is not acute disease episodes, but rather the ingestion of low level of toxins which may cause an array of metabolic, physiologic, and immunologic disturbances [3,4]. 

Although the literature about mycotoxins is rich in reports investigating cellular mechanisms, cellular toxicity, associated pathology and animal performance, studies on the effect of these compounds on the gastrointestinal tract (GIT) is limited. Considering the initial interaction of mycotoxins is with the gut epithelium, this topic has gained significant research interest in the last decade for a number of reasons. First, most acknowledge that a healthy intestinal tract guarantees the welfare and the health of both people and animals. Second, rapidly dividing and activated cells and tissues with a high protein turnover are predominant in gut epithelium. Intestinal cells and tissues can become a main target of mycotoxins as many of these metabolites are inhibitors of protein synthesis. Third, the absorption of mycotoxins and their fate within the GIT suggests that the epithelium is repeatedly exposed to these toxics, and at higher concentrations than other tissues. This latter point is further explored in the next section of this review.

The maintenance of a healthy GIT is crucial as it insures that nutrients are absorbed at an optimum rate, it provides efficient protection against pathogens through its own immune system, and it maintains the indigenous microflora in adequate numbers and confined to their natural niches. These three functions of the GIT might be pictured such as a “ménage à trois” in which each component interacts with each other (nutrition ↔ immune system ↔ gut microbiota ↔ nutrition) to maintain intestinal homeostasis. Whenever the integrity of the intestinal mucosa is compromised, nutrient absorption decreases. In addition, an increased proportion of absorbed nutrients is directed to repair the damaged area and to support the immune system until the intestinal insult is eliminated [5]. 

If one component from the “ménage à trois” is compromised, the adverse effect might be extended to the other components. For example, altering the numbers and species of GIT bacteria may affect the host’s ability to digest food and to stimulate the immune system. In this review we have therefore summarized recent findings following mycotoxin exposure on digestive and absorptive functions, intestinal defense, and microbiome composition. This review is focused on the major mycotoxins in terms of occurrence and toxicity, namely aflatoxins (AF), ochratoxin A (OTA), deoxynivalenol (DON), T-2 toxin (T-2), zearalenone (ZEA) and fumonisins (FB). Seven intestinal processes were investigated as shown in the Table 1. In addition, animal responses were evaluated according to the experimental doses used. Based on recent mycotoxin surveys [6,7,8], these doses were placed in three different categories: realistic, occasional and unrealistic doses (Table 2). This approach was applied to all results reported in this review.

## 2. Intestinal Absorption and Fate of Mycotoxins with the Gut

**Figure 1:**
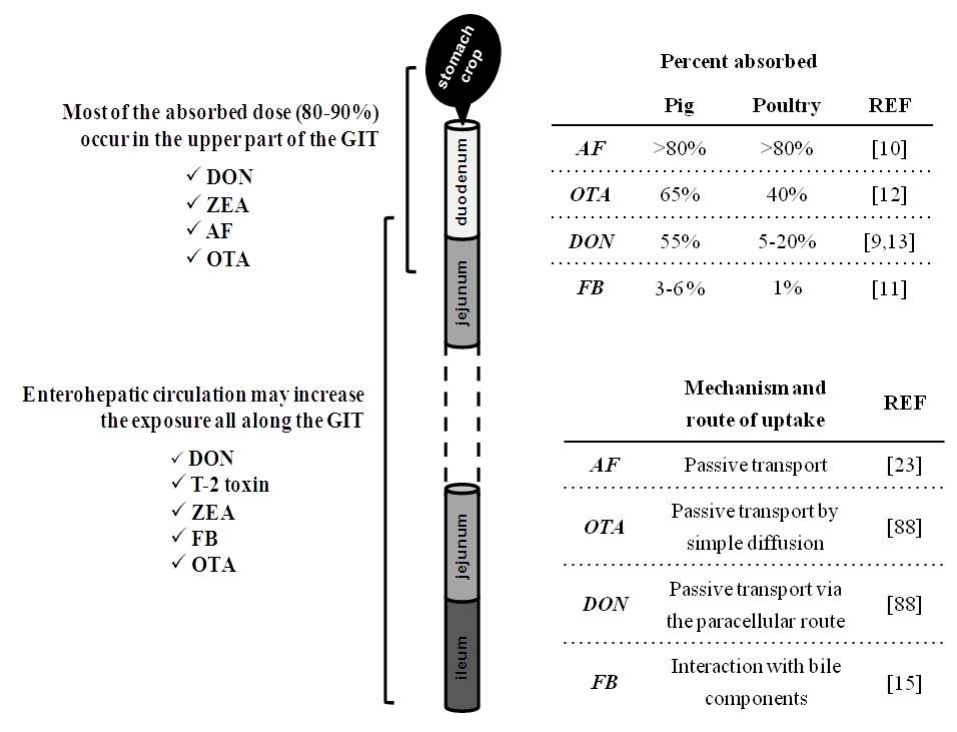
Absorption and fate of mycotoxins within the gastrointestinal tract (GIT) of non-ruminants. On the left are displayed the different segments of GIT, the sites of absorption and the dynamic of major mycotoxins within the GIT. It is a rough representation of the GIT of non-ruminants that does not take into account the size and proportion of these segments according to species. On the right is indicated the percent absorbed of major mycotoxins within the GIT of pig and poultry, and the routes of uptake of toxins.

Mycotoxin uptake and subsequent tissue distribution is governed by GIT absorption. This passage across the intestinal barrier may be maximal, as with aflatoxins (AF), or very limited, as with fumonisins (FB) (Figure 1). The bioavailability of these fungal compounds is thereby very diverse and differs between animal species (Figure 1). Regardless, the intestinal epithelium is exposed to the entire content of contaminated feed and is the first target of these contaminants. The rapid appearance of most mycotoxins in the circulation clearly indicates that the majority of the ingested toxin is absorbed in the proximal part of the GIT [9,10]. Mycotoxins can therefore compromise the intestinal epithelium either before absorption in the upper part or throughout the entire intestine by non-absorbed toxins. Indeed, with the exception of AF which is absorbed at high rates regardless of the species [10], absorption of other mycotoxins, such as trichothecenes (TCT), OTA, or FB may vary from 1% to 60% [9,11,12,13]. Thus, a substantial portion of non-absorbed toxin remains within the lumen of the GIT. The poor intestinal absorption of FB, ranging from 1% to 6% in non-ruminant species [11], implies that gut epithelium is exposed to a very high proportion of the toxin ingested. Similarly, absorption of deoxynivalenol (DON) is moderate in pigs, but very limited in poultry (Figure 1) [13,14]. The relative tolerance of poultry to DON has been partly attributed to its low bioavailability. However, the potential impact of the remaining DON in the intestinal lumen is still unknown, and the tolerance level within the GIT might be different. However, in comparison to pigs, the intestinal transit time is very rapid in poultry, and therefore this may reduce the exposure time to this mycotoxin. More importantly, several mycotoxins have been shown to undergo entero-hepatic circulation (Figure 1) [9,12,15,16].

**Table 1:** Intestinal processes investigated—number of experiments per process and per mycotoxin in the meta-analysis.

		Nutrient digestibility	Enzyme activities	Nutrient uptake ^1^	Digestive microflora	Barrier integrity	Mucosal immunity ^2^	Pathogen clearance	Total ^3^
Experiments		13	5	17	5	16	13	14	83
	* in vitro*/*ex vivo*/*in vivo* ^4^	0/0/13	0/0/5	1/10/12	1/2/4	13/2/5	7/1/10	1/1/13	23/16/62
	Aflatoxin (AF)	5	4	1	0	2	1	1	14
	Ochratoxin A (OTA)	0	0	0	0	3	0	3	6
	Deoxynivalenol (DON)	1	0	11	3	8	7	2	32
	T-2 toxin (T-2)	0	0	1	1	0	0	3	5
	Zearalenone (ZEA) ^5^	0	0	0	0	0	0	0	0
	Fumonisin (FB)	2	1	2	1	2	4	2	14
	Multi-contamination	5	0	2	0	1	1	3	12
References									
	Aflatoxin (AF)	[17,18,19,20,21]	[17,19,22,23]	[24]		[25,26]	[27]	[28]	
	Ochratoxin A (OTA)					[29,30,31]		[32,33,34]	
	Deoxynivalenol (DON)	[35]		[36,37,38,39,40,41,42,43,44,45,46]	[47,48,49]	[50,51,52,53,54,55,56,57]	[51,53,58,59,60,61,62]	[58,63]	
	T-2 toxin (T-2)			[64]	[65]			[28,66,67]	
	Fumonisin (FB)	[68,69]	[70]	[70,71]	[72]	[51,73]	[51,74,75,76]	[75,77]	
	Multi-contamination	[21,78,79,81]		[82,83]		[51]	[51]	[84,85,86]	

^1 ^Nutrient uptake includes also experiments on electrophysiological properties of the intestinal epithelium and on morphology of intestinal villi; ^2 ^Mucosal immunity does not include experiments using stimuli, such as pathogens or antigens, besides mycotoxin exposure. It refers mostly to the interaction of the mycotoxin with the epithelium through the analysis of cytokine expression; ^3 ^The same experiment may be assigned to different categories when multiple intestinal functions were investigated; ^4 ^*in vitro*, use of cell lines; *ex vivo*, use of isolated epitheliums or rumen simulation; *in vivo*, use of animals. Some published articles combined within the same study *in vitro* and/or *ex vivo* and/or *in vivo* approaches, especially for nutrition studies with the use of Ussing Chamber (UC) following animal intoxication. ^5^Although there is no report on ZEA alone, ZEA was found in many experiments using naturally contaminated feed with *Fusarium* toxins.

**Table 2:** Method used to categorize the experimental doses.

		Deoxynivalenol	T-2 Toxin	Zearalenone	Fumonisins	Aflatoxin	Ochratoxin A
		(DON; mg/kg)	(T-2; mg/kg)	(ZEA; mg/kg)	(FB; mg/kg)	(AF; mg/kg)	(OTA; mg/kg)
**Realistic doses (RD) ^1^**		<5	<0.5	<1	<10	<0.3	<0.3
	* Representative of field conditions*						
**Occasional doses (OD) ^1^**		>5	>0.5	>1	>10	>0.3	>0.3
	* Unfavorable weather conditions*	<25	<2	<5	<40	<2	<2
**Unrealistic doses (UD) ^1^**		>25	>2	>5	>40	>2	>2
	* Unlikely to occur in nature*						
**EU Limits (EC guidance) ^2^**							
	Pig (young)	0.9 (0.9)	no advisory or guidance levels established	0.25 (0.1)	5 (5)	0.02	0.05 (0.05)
	Poultry	5		-	20	0.02	0.1
	Ruminant (young)	5 (2)		0.5 (0.5)	50 (20)	0.02 (0.01)	-
**USA Limits (FDA guidance) ^3^**							
	Pig (young)	1	no advisory or guidance levels established	no advisory or guidance levels established	10	0.1 (0.02)	no advisory or guidance levels established
	Poultry (young)	5			50 ^4^	0.1 (0.02)	
	Ruminant (young)	5			30 ^4^	0.3 (0.02)	

^1 ^The establishment of the three categories (RD, OD, UD) was based on recent worldwide surveys [6,7,8]. According to the mean concentrations reported in these surveys, an average was set for each mycotoxin and multiplied by a 5-fold factor to get the maximum threshold for realistic doses (RD). According to the maximum levels detected in these surveys, an average was set for each mycotoxin and multiplied by a 2-fold factor to get the minimum threshold for unrealistic doses (UD). Occasional doses (OD) include concentrations between the two thresholds set for RD and UD. The conversion of doses used *in vitro* to the equivalent doses in mg/kg was based on the method of Sergent *et al.* [87]; ^2 ^EU limits in finished feed set according to the European Commission Recommendation 2006/576/EC and the European Commission Directive 2003/100/EC; ^3 ^USA limits in finished feed set according to the Food and Drug Administration Regulatory Guidance for Toxins and Contaminants; ^4 ^In animals fed for slaughter.

This makes the mycotoxins available again via the bile in the entero-hepatic cycle, resulting in reabsorption and a prolonged retention time in the GIT. Entero-hepatic recycling must contribute to FB toxicity as intestinal absorption of this toxin is very low. Thus, in the intestinal lumen FB could be incorporated into mixed micelles, through interactions with cholesterol and/or bile salts, and thereby facilitate its intestinal absorption [15]. Interestingly, an *in vitro* study showed that FB was unable to cross the intestinal epithelium [89], but considering this mode fails to mimic *in vivo* conditions (e.g., through entero-hepatic recycling or through incorporation into mixed micelles) conclusions must be drawn carefully. In addition to the increased exposure through entero-hepatic circulation, it has been recently suggested that DON first enters the blood circulation when absorbed in the upper GIT and then reenters the intestinal lumen, passing through the more distal intestinal cells from the blood stream via the basolateral side of the cell [50]. Although an *in vitro* study, this may explain why some effects of DON are observed in the mid and distal jejunum rather than the proximal intestine. 

Mycotoxin metabolism can occur in both the liver and the digestive tract. Intestinal metabolism, whether be in the gut epithelium or by gut microorganisms, may limit the toxic effects of mycotoxins within the GIT. This is especially true for ruminants which are able to convert many mycotoxins into non-toxic metabolites. Ruminant resistance to some mycotoxins has been attributed to the detoxifying role of the microbial population in the rumen. Many compounds, such as ZEA, DON or OTA are effectively rendered non-toxic by rumen microorganisms before absorption [9]. However in non-ruminants, intestinal biotransformation of mycotoxins takes place predominantly in the large intestine and thus provides little detoxification prior to absorption. Unlike other mycotoxins, AF requires metabolism to its toxic metabolite. While this activation has been largely described in the liver, AFB_1_ metabolism to the reactive epoxide also takes place in the intestinal tract [88]. 

The absorption and fate of mycotoxins within the GIT provides evidence that the intestinal epithelium is prone to the toxic action of these toxins. Therefore, this paper reviews the consequences of mycotoxin exposure on the different processes governing the major functions of the intestine.

## 3. Consequence of Mycotoxins for Nutrient Metabolism

It is very well documented that the major mycotoxins are able to adversely affect the growth of animals. Impact on performance varies according to many factors, such as the mycotoxin and the species used, the concentration in feed, the use of purified *versus* naturally contaminated feed, or the ingestion of multi-toxin contaminated feed. Although the effect of low doses is more controversial, the reduced performance is among the main characterized effects of mycotoxin intoxication [3]. Though decreased body weight gain of animals is seemingly a consequence of reduced feed intake or outright feed refusal, a significant body of research points to a direct and/or indirect effect of mycotoxins on the nutrient quality, digestibility and/or absorption. There is a close association between production performance and digestive activity. For example, authors from the 1970’s and 1980’s reported reduced absorption of essential nutrients after aflatoxin [3] and trichothecene (TCT) [36,64] exposure. A reduction in the severity of acute aflatoxicosis in broilers oversupplemented with energy has been noted [90]. Thus, it is important to elucidate how mycotoxins modulate the activity of enzymes and transporters involved in nutrient digestion and uptake, and subsequently, the consequences on nutrient digestibility and on metabolizable energy.

### 3.1. Nutrient Digestibility and Metabolizable Energy

The effect of mycotoxins on apparent nutrient and energy digestibility has been documented, especially in case of AF and *Fusarium* toxins (FUS). Very low doses of AFB_1_ (20 and 40 μg/kg) reduced the apparent digestibility of crude protein by 8% to 13% in ducks (Table 3) [17]. Similarly, dietary AF was suggested to increase the amino acid requirements, and also to affect ducks to a greater extent than chickens [18]. At higher doses, this *Aspergillus* metabolite showed similar effects on the apparent digestibility of laying hens and broiler chickens [19,20]. Net protein utilization, apparent digestible (ileal digesta) and metabolizable energy (excreta) were also evaluated in these studies, and AF was shown to reduce energy utilization (Table 3). 

**Table 3:** Modulation of digestive and absorptive processes by mycotoxins.

		MYCOTOXIN CONCENTRATION IN STUDIES		
		Realistic doses	Occasional doses	Unrealistic doses
Digestive processes				
	Enzyme activities	**AF** (hen): amlysase activity ↗ in pancreas and ↘ in duodenum, lipase activity ↘ in pancreas and duodenum, trypsin and chymotrypsin activity ↗ in pancreas [22]. **AF** (duck): protease, amlysase, trypsin and chymotrypsin activity ↗ in duodenum [17].	**FB_1_** (pig): aminopeptidase activity ↘ in jejunum [70]. **AF** (hen): disaccharidase, maltase activity ↗ in jejunum [19]. **AF** (mouse): alkaline phosphatase activity ↘ in isolated duodenal enterocytes [23].	
	Nutrient digestibility	**AF** (duck): reduced apparent digestibility of crude protein [17,18]. **FUS** (dog): improved nutrient digestibility [81]. **FB_1_** (pig): reduced digestibility of ether extract [68]. **DON** (chicken): reduced intestinal viscosity [35].	**FUS** (hen): slightly depressed nutrient digestibility & metabolizable energy [78]. **FUS** (chicken): increased protein digestibility & net protein utilization [80]. **FB_1_** (rat, pig): reduced nutrient digestibility [68,69]. **AF** (chicken/hen): reduced apparent digestibility, digestible & metabolizable energy [19,20,21].	
Absorptive processes				
	Sugar transport	**DON** (HT-29 cells): strong inhibition of SGLT1 & GLUT5 [44]. **DON** (chicken): reduced intestinal expression of SGLT1, GLUT2 [40] & GLUT5 [46].	**DON** (chicken-hen/UCj ^1^): reduced Isc after glucose addition [42,43], inhibition of intestinal SGLT1 [45 ]. **FB_1_** (pig/UCj ^1^): enhanced Isc after glucose addition [70].	**T-2 toxin** (rat/explant): reduced glucose absorption in jejunum and its rate of appearance in venous plasma [64]. **AF** (UCj ^2^): reduced Isc after glucose addition [24]. **OTA** (HT-29 cells): strong inhibition of SGLT1 [29].
	Amino-acid transport	**DON** (HT-29 cells): inhibition of active and passive L-serine transporters [44].	**DON** (UCj ^2^): reduced Isc after proline addition [41].	
	Lipid transport	**DON** (HT-29 cells): increase of palmitate transport [44]. **DON** (chicken): reduced expression of palmitate transporter in jejunum [46].		
	Other essential nutrients		**DON** (mouse/explant): reduced uptake and transfer of folate [36].	

GLUT2, facilitated glucose transporter; GLUT5, fructose transporter; Isc, short-circuit current; SGLT1, sodium-dependent glucose cotransporter 1; UCj, jejunum mounted in Ussing Chamber. ^1 ^(species/UCj) means trials with animals exposed to mycotoxins through feeding or gavage, followed by jejunum mounted in Ussing Chamber; ^2 ^(UCj) means addition of mycotoxin on the mucosa of jejunum mounted in Ussing Chamber from non-exposed animals.

In addition, AF in combination with OTA had a more pronounced effect on metabolizable energy content of the diet than when either toxin was fed alone, and this reduction occurred through a significant increase in the maintenance energy requirement of the hen [21]. The inability of animals to efficiently utilize the essential nutrients in their feeds has also been demonstrated in rats and pigs fed contaminated diets with moderate doses of FB_1_ (Table 3) [68,69]. As mentioned by Gbore *et al.* [69] the alterations in albumin synthesis and serum protein concentrations observed sometimes in intoxicated animals might be a consequence of a lower protein digestibility. Other studies related to the effects of DON and FUS (primarily contaminated with DON) on nutrient digestibility show how relevant it is to consider the evaluation of naturally contaminated grains. Although some authors did not find any, or only minor differences, in nutrient digestibility [78,79,82], Danicke *et al.* [80] and Leung *et al.* [81] reported that diets naturally contaminated with FUS improved the digestibility in broilers and dogs respectively (Table 3). Similarly, DON-contaminated feed has been shown to reduce the intestinal viscosity of broilers, probably due to a direct effect on the content of non-starch polysaccharides (considered as anti-nutritive) in the feed (Table 3) [35]. A plausible explanation is that growing fungi are capable of synthesizing cell wall degrading enzymes in order to penetrate the cell wall of cereal kernels. This partial degradation of the cell wall constituents and the structural changes in the protein and probably in other nutrient fractions, would suggest an improvement in nutrient availability for the animal. 

These results stress the necessity to consider not only the mycotoxin contamination in evaluating their effect on animal health and performance, but also the possible physicochemical alterations of feedstuffs due to the infection or invasion of the fungus. Nonetheless, inconsistent results on animal performance has been reported in these studies, ranging from decreased body weight [80,81] but with a better feed-to-gain ratio [80], to increased animal growth [35]. Also, other suggestions, such as physiological adaptations, have been proposed to explain higher digestibility. Reduction of feed intake minimizes mycotoxin exposure that would result in lesser amounts of bulk passing through the GIT, thereby increasing nutrient digestibility and absorption [81]. Yunus *et al*. [82] reported a tendency of higher protein digestibility in broilers fed a diet containing DON. However, the authors of this study did not use naturally contaminated grains, and thereby suggested that the increase observed in the length of the small intestine might be responsible for the improvement in digestibility. These physiological changes would imply a higher absorption of mycotoxins, and would account for the decreased performance observed in animals.

### 3.2. Digestive and Absorptive Processes

The measure of apparent digestibility reflects the net effect of all digestive and absorptive processes along the digestive tract. Accordingly, attention needs to be paid to the effect of these fungal metabolites on the individual components of these processes and endogenous losses of both nutrients and energy. 

#### 3.2.1. Activity of Digestive Enzymes

Digestive enzymes are required for the digestion of dietary starch, fat, and proteins. Disturbances to enzyme production and/or activity may lead to GIT disorders. Similar to the studies on nutrient digestibility, several reports have concluded that enzyme activity is modulated following AF consumption. Contradictory effects were reported in birds fed realistic concentrations of AF, on the duodenal activity of amylase (Table 3) [17,22]. Despite that, both authors agree on the plausibility of pancreatic damage, resulting in increased release of proenzymes from pancreatic cells to the intestinal tract. This would account in their studies for the higher activity of amylase, trypsin and chymotrypsin in both the pancreas and duodenum (Table 3) [17,22]. However, digestion of nutrients was not enhanced in the intestine [17]. Applegate *et al.* [19], suggested that a compensatory response to a decrease in feed intake and nutrient deficiency during aflatoxicosis might also explain these findings. 

Increased jejunal activity of disaccharidase and maltase was also noted with higher doses of AF in hens (Table 3) [19]. Conversely, alkaline phosphatase and aminopeptidase activity were reduced in the intestine of mice orally treated with AF and of pigs fed FB_1_-contaminated feed, respectively (Table 3) [23,70]. These changes in activity may reflect changes in intestinal villi morphology as discussed below. It is known that the upper 40% of the villus expresses 30% to 40% more sucrase and maltase activity per enterocyte than the lower 60% of the crypt villus axis. Therefore, enterocytes must differentiate during their time along the crypt-villus axis to fully express these digestive functions [19]. 

#### 3.2.2. Morphology of Intestinal Villi

Nothing is known about mycotoxin effect on enterocyte differentiation or migration rates along the length of the villus, but many studies reported adverse effects of these fungal metabolites on morphology of intestinal villi. Villi increase the internal surface area of the intestinal walls, allowing for an increase of the area available for nutrient absorption. Therefore, whenever integrity of the intestinal wall is compromised, the effectiveness of nutrient absorption might be affected. In the pig, exposure of the GIT epithelium to moderate doses of FB reduced villi height and caused villus fusion and atrophy [71,74]. At low doses of DON, the same villi abnormalities were noted in the jejunum of pigs [37,51]. Similarly in poultry, either low or moderate levels of DON in feed, as well as its combination with other *Fusarium* toxins were able to lower the absorptive surface area through a decrease of villus height in the duodenum and jejunum [38,39,40,82,83,84]. Since DON is an inhibitor of protein synthesis, it is not surprising to see the mucosal structure altered following toxin ingestion. Indeed, villi and crypt are intestinal areas with a high rate of protein turnover. 

#### 3.2.3. Nutrient Uptake

**Figure 2:**
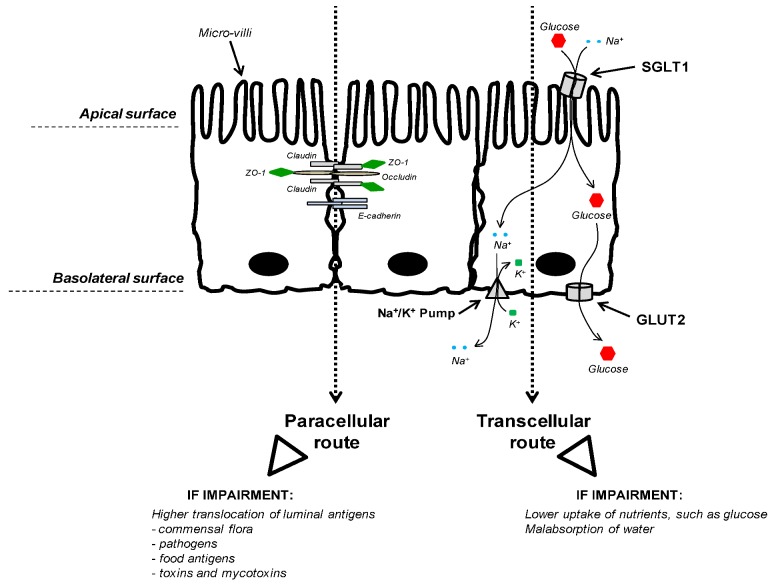
Intestinal Epithelial Cells (IECs)—transcellular and paracellular pathways. The figure displays three enterocytes from an epithelium of the small intestine. The side of the epithelial tissue facing the lumen is the apical surface, and the surface that adjoins underlying tissue is the basolateral surface. The left side of the figure exemplifies the tight junction (TJ) complex involved in the paracellular route of absorption. TJ are the closely associated areas, at the apical side, of two cells whose membranes join together forming a paracellular barrier. It is a rough representation of the TJ complex, including only the proteins claudin, occludin and ZO-1. In addition, E-cadherin also participates to cell adhesion. The right side of the figure exemplifies the glucose transport through the transcellular route of absorption. The main apical transporter for active glucose uptake in small intestine is the sodium-dependent glucose cotransporter 1 (SGLT1). SGLT1 couples the transport of two Na^+^ ions and one glucose molecule to mediate unidirectionally glucose absorption from the intestinal lumen into epithelial cells. This symporter uses the electrochemical gradient of Na^+^ to drive the glucose absorption. The basolateral transporter GLUT2 (facilitated glucose transporter) facilitates diffusive transport of intracellular glucose into bloodstream.

To investigate the effects of mycotoxins on nutrient uptake and transport, several studies have utilized the Ussing Chamber (UC) method. The UC is used for electrophysiological studies of all epithelial tissues, and some of the parameters that can be investigated are transepithelial electrical potential or short-circuit current (Isc). This latter measure, Isc, is induced by the absorption of sodium (Na^+^) and the secretion of chloride (Cl^−^) ions. Measurement of Isc is a good indicator of sugar or amino acid transport as many nutrients are transported by carrier systems, and are usually cotransported with Na^+^ (Figure 2). Thus, if those nutrients are added to the mucosal side of intestinal tissues, carrier-mediated transport is stimulated with a concomitant rise in the uptake of Na^+^ [41]. 

Awad and co-workers generated substantial data on electrophysiological properties of intestinal epithelium following either the feeding of animals with DON-contaminated diet or the direct exposure of this mycotoxin to the epithelium [41,42,43]. In both cases, the UC was used to examine the bird’s jejunum. Addition of glucose [42,43] or L-proline [41] on the luminal side of the isolated mucosa increased Isc, indicating that this induction was due to increased Na^+^ cotransport. This effect was reversed by moderate doses of DON (Table 3). Inconsistent effects of AF on glucose-induced Isc were reported with different *in vitro* concentrations of AF [24]. By contrast, FB_1_ has been shown in pigs to enhance Isc after glucose addition (Table 3) [70]. It appears that the effects on the electrophysiological response are most likely a result of mycotoxin modulation on Na^+^ cotransport. 

Maresca *et al.* [29] were the first to show *in vitro* that OTA decreased glucose absorption mediated by the active Na^+^-dependent glucose transporter SGLT1 (Table 3). A year later, the same group demonstrated that low concentrations of DON inhibited the uptake of substrates with specific intestinal transporters (Table 3) [44]. They concluded that this was a specific modulation of the activity of intestinal transporters rather than a consequence of non-specific cell damage by the toxin. SGLT1 appeared to be the most DON-sensitive transporter, followed by the passive fructose transporter GLUT5 [44]. In addition, passive and active L-serine transporters exhibited a moderate sensitivity, whereas passive sugars transporters of the GLUT family were only slightly affected by the mycotoxin [44].

Considering glucose is a key fuel and an important metabolic substrate in animals, further work investigating the direct effects of DON on glucose transporters (Figure 2) has been reported. When added on the luminal side of the jejunum, DON mimicked the effect of a specific SGLT1 inhibitor, resulting in decreased glucose uptake [45]. Recently, gene expression of SGLT1 and GLUT2 (facilitated glucose transporter) were also evaluated after DON exposure (Table 3) [40]. The study revealed that the mRNA level of these genes was very low in the small intestine of chickens, especially in the proximal part, suggesting that down-regulation contributes to the inhibitory effect of DON on intestinal glucose absorption. The effect on GLUT2 was less obvious, which is in agreement with Maresca *et al.* [44]. Since this transporter mainly mediates the basolateral exit of glucose, unlike SGLT1 anchored on the apical membrane (Figure 2), GLUT2 would not be as exposed to DON. In summary, the decreased absorption of glucose observed following DON [36,45] or T-2 toxin intoxication [64] is consistent with a direct effect on SGLT1. In addition to this anti-nutritional effect, inhibition of SGLT1 could also cause diarrhea since this transporter is responsible for water reabsorption. 

### 3.3. Connection between Intestinal Nutrient Metabolism and Animal Growth

**Figure 3:**
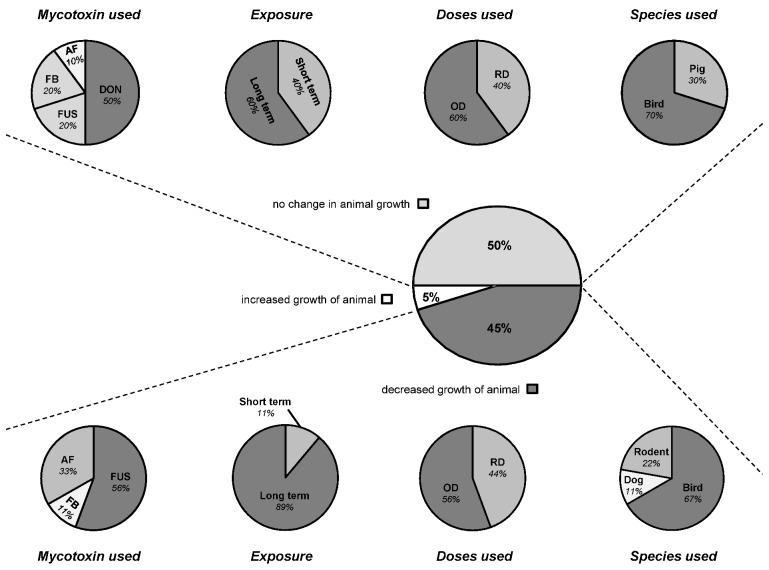
Modulation of digestive and absorptive processes by mycotoxins—Consequence for animal growth. The figure displays animal body weight changes when an effect was reported on nutrient digestibility and/or nutrient uptake and/or intestinal morphology. These charts refer to 18 published articles [17,19,20,23,35,38,39,40,42,51,69,70,74,78,80,81,82,83] but reflect 20 separate studies since two articles used different concentrations of toxins within their study. The central and prominent chart refers to the overall modulation of growth balance by mycotoxins via digestive and absorptive processes. Half of these studies (10/20) did not observe changes in animal growth, 45% (9/20) observed a decrease in animal growth, and 5% (1/20) noted increased growth of the animal. According to the effect observed on animal growth, four sub-charts were established according to the cited experimental design, namely the mycotoxin used, the duration of exposure, the doses used and the species used. Short term exposure refers to trials of less than three weeks of duration, and long term to trials of more than three weeks exposure. RD, Realistic Doses; OD, Occasional Doses.

Given the effects of mycotoxins on the diverse processes of digestion and nutrient uptake, it is reasonable to ascribe the adverse effect observed on animal growth to the impairment of these processes. Nonetheless, as depicted in Figure 3 animal growth is often not or only moderately affected following the modulation of digestive functions by mycotoxins. Figure 3 summarizes the outcomes from 20 studies on animal body weight gain when authors reported any significant effects on nutrient digestibility, enzyme activity, nutrient uptake and transport, as well as on intestinal morphology. This summary allows an overview of the contribution of these digestive changes on animal performance. Half of the studies did not observe any changes on body weight whereas nine studies reported decreased growth. As previously mentioned, one study noticed an improvement in animal growth [35].

Interestingly, further analysis of these studies show that animal growth was mostly impaired when DON was in combination with other Fusarium toxins (FUS) (predominantly contaminated with DON) compared when given alone in the feed (Figure 3). In these experiments, species fed with DON or FUS contaminated diets were mainly broiler chickens and laying hens (Figure 3). Poultry are known to be relatively tolerant to DON up to 15 mg/kg, but at lower doses of DON in the FUS diets a high sensitivity was noted. Due to the natural co-occurrence of DON and ZEA, this latter was the second most important metabolite found in these studies. Similarly very high concentrations of ZEA are not seemingly detrimental in birds, but its combination with DON may exacerbate the DON effect and greatly impact animal health and productivity. Furthermore, Pinton *et al*. [52] demonstrated very recently that 15-ADON, an acetylated derivative of DON commonly produced together with DON, caused higher toxicity than DON in the intestine. These findings emphasize that multi-toxin contamination should be further considered in the evaluation of the toxicity [91]. Figure 3 shows that a long-term trial, recreating farm conditions, is more suitable to fully describe the effects of mycotoxins, especially at low doses. On the other hand, as animal growth was not always affected, compensatory mechanisms must take place in animals to counterbalance the anti-nutritive effects induced by mycotoxins. For instance, increased absorption in distal intestinal sites to compensate for reduced nutrient absorption in the proximal intestine could be one of them [40]. Girgis *et al*. [84] observed an increase of villus height in the jejunum and ileum of birds fed a contaminated diet with Fusarium mycotoxins. The authors suggested that this finding may represent compensation for the reduced surface area of the duodenal villi resulting from reduced villi heights in these birds. 

To conclude, evidence suggests that animal growth can be impaired regardless of the modulation observed on digestive and absorptive processes. Dramatic effects were seen on feed consumed and live weight gain whereas no difference was observed in apparent nutrient digestibility after DON feeding in pigs [79]. Thereby, reduced feed intake seems to mainly contribute to the reduced weight gain observed in animals. A very recent study showed that aberrant release of gut satiety hormones (in response to toxic substances to diminish and prevent further ingestion of the agent) might be one critical underlying mechanism for DON-induced anorexia and ultimately growth suppression [92]. Very recently, Pastorelli *et al*. [93] showed that the contribution of the reduced feed intake to the reduction of the body weight gain in pigs was more than 70% for mycotoxicoses. The authors reviewed the consequences of different sanitary challenges (digestive bacterial infections, poor housing conditions, lipopolysaccharide (LPS) challenge, mycotoxicoses, parasitic infections and respiratory diseases) on feed intake and growth responses of pigs. For challenges associated with the gastrointestinal tract, a large part of the reduction in growth was due to an increase in maintenance requirements, suggesting digestive and metabolic changes (repair of damaged tissues, maintenance of the integrity of the GIT, as well as metabolic cost associated with the stimulation of the immune system). It would be worthwhile reevaluating this partitioning in case of co-exposure to both a mycotoxin and a digestive pathogen. In the next section of this review, the potential of mycotoxins to enhance the toxic effects of intestinal pathogens is highlighted. 

## 4. Consequence of Mycotoxins on Intestinal Defense

The gastrointestinal tract (GIT) possesses its own immune system and it is estimated that up to 70% of the immune defenses of the organism are located in the intestine. In the GIT, the mature immune system of animals consists in specific tissues, such as gut-associated lymphoid tissues (GALT; Peyer’s patch, mesenteric lymph nodes, cecal tonsils) where immunocompetent cells are able to mount an efficient immune response. Complementary to that, early and immediate responses are provided locally along the length of intestine, where mucus, intraepithelial immune cells as well as intestinal epithelial cells (IECs) play a key role as sentinels and defenders. 

Though it is well accepted that mycotoxins are able to modulate immune responses [4], the consequence of this immunomodulation for the GIT has been less documented. The capacity of animals intoxicated with mycotoxins to regulate the intestinal immune balance and/or mount an appropriate intestinal immune response is addressed here. 

### 4.1. Pathogen Clearance

The GIT is a major portal for entry of most enteric pathogens and is also a common route for vaccination in poultry. Intrusion of pathogens or antigen delivery for vaccination induces activation of the intestinal immune system, resulting in the division and proliferation of immune cells. As previously mentioned, actively dividing cells are the main targets of mycotoxins, and therefore feeding animals with mycotoxin-contaminated diets can lead to greater susceptibility to enteric infections. Table 4 presents results from studies where animals, exposed to mycotoxins, were not able to efficiently control different pathogen infections and clear them from the intestine.

#### 4.1.1. Parasitic Infections

Coccidiosis is probably the most common disease in modern poultry production. It is caused by protozoan parasites of the genus Eimeria. These are obligate intracellular parasites with complex life cycles including sexual and asexual stages. In poultry, Eimeria affect the intestine making it prone to other diseases (necrotic enteritis) and reducing the ability of this organ to absorb nutrients. At high doses of OTA (2–4 mg/kg), coccidiosis provoked by *E. acervulina* and *E. adenoeides* in chicks and turkey poults respectively can progress more strongly and rapidly in OTA-treated animals than in those not exposed to the mycotoxin [32,33]. Lesion and oocyst indexes in the intestine of animals fed OTA were higher, and mucosa damage was more intense (Table 4). In addition, earlier mortality was also noticed in these studies. Using an optimized mixture (inducing lesions without mortality) of *E. acervulina*, *E. maxima* and *E. tenella*, or only *E. maxima*, Girgis *et al*. [84,85,86] examined the impact of the parasitic disease in the intestine of chickens fed with grains naturally contaminated with multiple *Fusarium* mycotoxins. DON was inevitably the major contaminant in the grains with concentrations representative of field conditions in North America (3.8–6.5 mg/kg). Along with DON, 15-acetyl DON and ZEA were present in the grains. Although a concentration up to 15 mg/kg of DON is regarded as safe in poultry, the lower doses used in these studies and their potential interaction with the other *Fusarium* mycotoxins, interfered with intestinal recovery and modulated intestinal immune response to coccidial infections (Table 4). Clearance of the parasitic infection is known to be dominated by Th-1 responses through recruitment and stimulation of lymphocytes at the site of coccidial infection. Feed-borne *Fusarium* mycotoxins lowered the percentage of CD4^+^ and CD8^+^ cells in jejunum of birds following a primary inoculation of *E. maxima*, suggesting a delayed response or an inhibition in the recruitment of these cells [86].

**Table 4:** Modulation of intestinal defense by mycotoxins during pathogen exposure.

	Microorganism in Contact with the Intestinal Epithelium		
	Parasite	Bacteria	Virus
Realistic doses ^1^			
	**FUS** (chicken): impaired recovery of duodenal villi from coccidial lesions [84], upregulation of IFN-γ expression in CT [85].	**FB_1_** (pig): increased intestinal colonization by *E. coli* [77].	
		**DON** (porcine cells & ileal loop): enhanced *S. typhimurium* invasion and translocation, potentiation of pro-inflammatory cytokines [58].	
Occasional doses ^1^			
	**FUS** (chicken): delayed recruitment of CD4^+^ and CD8^+^ cells in jejunum [86].	**FB_1_** (pig): longer shedding of *E. coli*, reduction of *in vivo* APC maturation (MHC-II, IL-12p40), T cell stimulatory capacity, specific Ig in PP [75].	
Unrealistic doses ^1^			
	**OTA** (turkey, chicken): bloody diarrhea, higher lesions and oocyst in intestine [32,33], duodenal hemorrhages [32].	**OTA** (chicken): higher number of *S. typhimurium* in duodenum & cecum, acute enteritis [34].	**T-2** (mouse): inability to clear reovirus from intestine, increased fecal shedding of the virus, suppression of IFN-γ expression in PP [67].
			**DON** (mouse): increased fecal shedding of reovirus, elevated intestinal virus-specific IgA, suppressed Th1 & enhanced Th2 cytokine expression [63].

APC, Antigen-Presenting Cells; CT, Cecal Tonsils; MHC-II, Major Histocompatibility Complex class II molecules; PP, Peyer’s Patches; ^1 ^Findings reported in the table refer to the outcomes on animals exposed to mycotoxins and challenged with microorganism compared to animals non-exposed and challenged with microorganism. Are only presented results at the intestinal level, systemic results were voluntarily omitted. This allows finding out on the potential of mycotoxins to exacerbate the intestinal response facing pathogens.

Migration of these types of cells from peripheral blood to the intestine might replenish these subsets in the jejunum, and could therefore account for the unchanged cell population following the secondary inoculation of *E. maxima* [86], and/or for the decrease in the percentage of these subsets in the blood of challenged birds [85]. In addition, the mRNA level of IFN-γ was up-regulated in cecal tonsils (chicken lymphoid tissue belonging to the GALT) of challenged birds fed the contaminated diet in comparison to the birds fed the control diet [85]. IFN-γ expression is related to increased resistance to coccidia and lowered oocyst yield during primary infections. However, no effect was observed on oocyst counts. Interestingly, Varga and Vanyi [66] demonstrated that the effectiveness of lasalocid (a coccidiostat) was impaired when the levels of T-2 toxin exceeded 0.5 mg/kg in feed, as depicted by the development of clinical coccidiosis in birds.

#### 4.1.2. Digestive Bacterial Infections

*Salmonella* is considered as a threat in the poultry industry not because of the serotypes specific for poultry, but for the serotypes that are carried most of the time asymptomatically in poultry (mostly *Salmonella enterica* serovar Enteritidis or *S. enterica* serovar Typhimurium) and cause food-borne illnesses in humans. It is because of the huge impact these bacteria have on public health that several countries are regulated to reduce *Salmonella* contamination in eggs and chicken carcasses. Numerous factors can affect the susceptibility of chickens to *Salmonella* colonization, including: age, stress, the genetics of the chicken, as well as mycotoxins. Chickens orally administered with a high dose of OTA (3 mg/kg) exhibited significant numbers of *S. typhimurium* in the duodenal and cecal contents when compared to non-administered birds [34] (Table 4). By contrast, feeding chicks with high levels of AF or T-2 toxin has no effect on incidence or severity of *S. typhimurium* colonization [28]. As pigs can also be a carrier and subsequently a contaminating source for the environment or for carcasses, the interaction of *S. typhimurium* with low doses of DON (equivalent to 0.9 mg/kg) was examined through *ex vivo* and *in vitro* approaches [58] (Table 4). Porcine ileal loops were used to reproduce *S. typhimurium* induced intestinal inflammation. When given separately, DON and *S. typhimurium* had no or minor effects after 6 h on the expression levels of cytokines and chemokines. Conversely, the co-exposure showed that DON dramatically enhances the inflammatory response to *S. typhimurium* in the ileal loops, with a clear potentiation of the expression of IL-1β, IL-8 or IL-6. As suggested by the authors, this potentiation coincided with a significantly enhanced Salmonella invasion in and translocation over intestinal epithelial cells, exposed to non-cytotoxic concentrations of DON for 24 h.

A higher susceptibility of the GIT to bacteria other than *Salmonella* was also reported in pigs treated with FB_1_ [75,757] (Table 4). Indeed, two separate studies analyzed the effect of low to moderate doses of FB_1 _(5 to 15 mg/kg) for 6-10 days on intestinal colonization and mucosal response to pathogenic strains of *Escherichia coli* (ETEC, Enterotoxigenic *E. coli* and ExPEC, Extraintestinal pathogenic *E. coli*). The prolonged intestinal infection observed by Devriendt *et al*. [75] is in accordance with the increased intestinal colonization reported by Oswald *et al*. [77]. Besides, translocation of bacteria to the mesenteric lymph nodes and dissemination to the lungs, and to a lesser extent to liver and spleen were observed in FB_1_-treated pigs in comparison to untreated animals [77]. In addition to the longer shedding of *E. coli*, Devriendt *et al*. [75] also showed that FB_1_ was able to reduce the induction of an antigen-specific intestinal immune response following oral F4 fimbriae (surface protein of ETEC) immunization. They demonstrated that many steps required in the establishment of an efficient immune response were affected in the intestine of animals treated with FB_1_. The T cell stimulatory capacity and the production of intestinal specific Ig were impaired, most likely due to the effects on antigen-presenting cells (APC). APC have a pivotal role in the mucosal immune system by connecting innate and acquired immune responses, through uptake of antigen in the lamina propria, maturation and migration to GALT, and interaction in these areas with T cells. In their study, the reduced upregulation of MHC-II, CD80/86, and IL-12p40 expression might account for the low response of intestinal APC from FB_1_-exposed piglets to F4 stimulation. As a consequence of the impairment of APC maturation, these cells were no longer able to efficiently interact and stimulate intestinal T cells, leading eventually to a defective production of specific Ig. As noted by Oswald *et al*. [77], ExPEC under normal conditions can persist in the large intestine of pigs but is able to colonize the gut and translocate to internal organs when the immune system is compromised. The impaired immune response observed after FB_1_ exposure [75,76] would strongly account for the translocation of ExPEC to lungs, liver and spleen [77]. Regarding other bacteria, it has also been demonstrated in poultry that increasing DON concentrations reduced the capacity of cecal tonsil cells to engulf killed Staphylococcus aureus [59]. 

#### 4.1.3. Enteric Viral Infections

Mucosal immune response to enteric virus has been investigated in animals exposed to trichothecenes (TCT) [63,67] (Table 4). The doses used in these studies are unlikely to occur under field conditions and mice were used. Rodents are relatively resistant to mycotoxins, and therefore, the effects observed may occur at lower doses in sensitive species, such as the pig. In the two studies, an enteric reovirus infection was reproduced since this virus is considered a valuable model for investigating mucosal immunotoxicity. Both trials drew the same conclusion; a single exposure to either DON or T-2 toxin suppressed the host response to reovirus as evidenced by the inability to clear the virus from the intestine (especially with T-2 toxin) as well as by increased fecal shedding of the virus. This latter finding could enhance virus dissemination among individuals. As suggested by the authors, this suppression of the host response appeared to be related with a decreased expression of IFN-γ in Peyer’s Patches (PP). This observation that both DON and T-2 toxin inhibited IFN-γ expression early during the infection is consistent with a diminished clearance of reovirus infection and a suppressed Th1 response. As previously mentioned, IFN-γ facilitates antiparasitic or antiviral immunity by suppressing pathogen replication and by activating macrophages. In Li *et al*. [63], DON promoted a Th2 response through increased IL-4, IL-6 and IL-10 expression in PP, elevating reovirus-specific IgA and IgG responses. By contrast, T-2 toxin in the report of Li *et al*. [67] did not induce a similar robust effect on Th2 cytokines, and also suppressed the mucosal IgA response. Interestingly, this suppression after T-2 exposure might account for the less efficient clearance of reovirus in comparison to DON. In the two studies, a dose-response assay revealed that doses more representative of field conditions were sufficient to increase the viral RNA in PP or feces. Evaluation of these lower doses on the other parameters investigated would have been interesting.

### 4.2. Mucosal Immunity—Cytokine Balance

**Figure 4:**
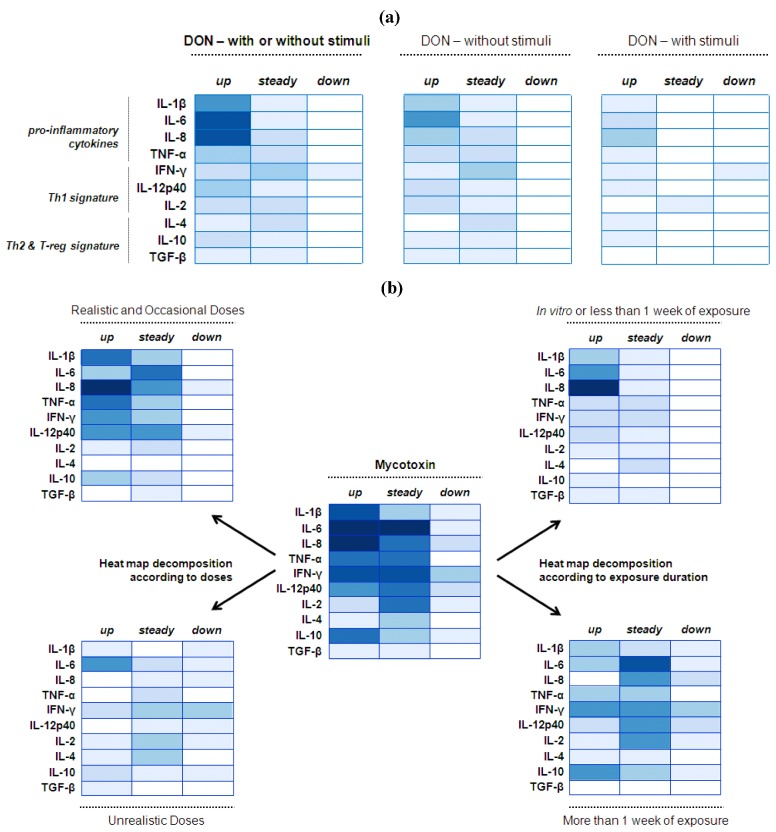
Modulation of intestinal cytokine balance induced by mycotoxins: Heat map representation. (**a**) Heat maps reporting DON modulation on intestinal cytokine balance; (**b**) Heat maps reporting mycotoxin modulation on intestinal cytokine balance. R software [94] was used to establish the heat maps. Here, this graphical representation reports the number of published studies reporting either up or steady or down regulation of certain cytokines. The values (here the number of published studies) are contained in a matrix that are represented by colors in place of numbers. The type of color used here is a spectrum of blue intensity. The more studies reporting the same effect for the same cytokine there is, the darker the cell will be. The maximum value is 7 and the minimum is 0. Example: a study reporting the up-regulation of IL-6 is numerically converted to a value of 1. Therefore, if IL-6 was shown to be up-regulated in five different studies, the value of IL-6 in the heat map would be as 5 and the blue intensity would be darker. By contrast, if IL-6 was shown only up-regulated in one study, the value in the heat map would be as 1 and the blue intensity would be lighter. Whenever authors reported an effect—up or down, or no effect—steady, on one cytokine, a value of 1 was attributed for this cytokine and categorized according to the effect noted. Figure 4a: These heat maps refer to 7 published articles [51,53,58,60,61,62,63] but reflect 10 separate studies if we consider that within the same article some authors assessed either the DON effect alone or combined with stimuli (pathogen or antigen). In line with that, the heat map on the left was therefore split in two sub-heat maps according to the exposure of the intestine to either DON alone or combined with stimuli. Among the 7 published articles, 2 studies analyzed DON effect *in vitro* on intestinal cell lines, 1 study analyzed DON effect *ex vivo* within ileal loops, and 4 studies analyzed DON effect *in vivo* on animals. Figure 4b: These heat maps refer to 13 published articles, the 7 previously mentioned on DON, plus 6 articles on mycotoxins other than DON [27,67,74,75,76,85]. However, some authors reported the separate effect of different mycotoxins within the same article, and/or with or without stimuli as well. Consequently, the heat maps were established according to 20 separate studies. Among the 13 published articles, 2 studies analyzed toxin effects *in vitro* on intestinal cell lines, 1 study analyzed toxin effect *ex vivo* within ileal loops, and 10 studies analyzed toxin effect *in vivo* on animals. Among the 13 published articles, 7 studies analyzed the effect of DON, 4 studies analyzed the effect of FB, 1 study analyzed the effect of AF, 1 study analyzed the effect of OTA, 1 study analyzed the effect of T-2 toxin, and 2 studies analyzed the effect of multi-contamination.

Cytokines are key signals in the intestinal immune system, and play pivotal roles in host defense, inflammatory responses, and autoimmune disease. These small peptide proteins, produced mainly by immune cells, facilitate communication between cells, stimulate the proliferation of antigen specific effector cells, and mediate the local and systemic inflammation in an autocrine, paracrine, and endocrine pathways. We thus paid particular attention to the cytokine balance following the exposure of the epithelium to mycotoxins (Figure 4a,b). The method used to examine the cytokine balance was based on the establishment of heat maps, and is detailed in the figure legend. 

#### 4.2.1. Deoxynivalenol (DON) Interaction with the Gut Epithelium

The effect of DON on cytokine balance was examined separately considering the significant amount of data generated for this mycotoxin. The heat map on the left clearly shows that exposure to DON led to an up-regulation of cytokine levels, especially the pro-inflammatory cytokines (Figure 4a). Many studies report an effect on IL-6, IL-8 and IL-1β [51,53,60,61]. Interestingly, down-regulation was only observed in one study and for IFN-γ. As previously mentioned this inhibition might interfere in the development of anti-viral responses [63]. 

To further investigate the effect of DON on the intestinal epithelium, the heat map on the left was subdivided into two complementary heat maps (Figure 4a). This decomposition allows separating studies using into either DON alone or combined with stimuli (pathogen or antigen). In the studies co-exposing the epithelium to both DON and stimuli, the resulting profile of cytokine expression might be attributed to a potentiation of the stimuli effect by DON [58,63] and not due to a direct effect of DON [58]. However, as shown in Figure 4a, DON itself is able to cause intestinal upregulation of pro-inflammatory cytokines. It is noteworthy that DON, as a protein synthesis inhibitor used at concentrations where it diminished metabolic activity, increases cytokine synthesis and secretion. It has been frequently observed that usually transient genes are overexpressed when using a protein synthesis inhibitor. This phenomenon is generally referred to as superinduction [61].

#### 4.2.2. Mycotoxin Interaction with the Gut Epithelium

Similarly, every study reporting cytokine expression in the GIT was analyzed in order to establish a heat map regardless of the mycotoxin used (Figure 4b). Overall, many authors observed an intestinal elevation of pro-inflammatory cytokines, and also of IFN-γ and IL-10. On the other hand, these cytokines, mostly the ones involved in Th1 response, remained unchanged in the small intestine of animals. Thus, it might be suggested that only certain cytokines are upregulated, or some mycotoxins had very few effects or no effect on gene expression [27,58,75]. However, the analysis itself should be considered as many authors reported only results for 1 or 2 target genes in their study [27,53,60,85] whereas others have analyzed a wider array of genes [51,58,74,75]. 

To further determine mycotoxins’ effect on the cytokine balance, the central heat map was decomposed using two parameters (Figure 4b). First, the heat map was subdivided into two complementary heat maps depicting the modulation of cytokine expression according to the doses used (Figure 4b). Evidence is displayed here that the increase in cytokine expression was mostly observed after exposure to low or moderate doses of mycotoxins. Although less studied, high concentrations of mycotoxins induced down-regulation of some cytokines, especially IFN-γ [74]. This tendency to repress gene expression could be related to cellular toxicity; induction of apoptotic cell death is commonly noticed at high toxin concentrations. Interestingly, IL-6 was still upregulated with high doses of toxins. This finding is attributed to exposure to DON which is seemingly able to induce this interleukin across a range of doses [51,61,62,63]. In the case of DON, it has been suggested that increased IL-6 expression induced the secretion of mucosal and systemic IgA, one of the most prominent features of exposure to this mycotoxin [95]. Secondly, a comparison was made between short and long term exposure to mycotoxins (Figure 4b). Short term exposure refers mainly to acute exposure through the use of *in vitro* or *ex vivo* models [53,58,60]. The differences between the two sub-heat maps are less obvious, with for instance upregulation still occurring in both conditions. However, the unchanged expression observed in chronic exposure might be a consequence of the early cytokine peaks observed in the short term trials. Indeed, the high expression level observed in the first hours or days could come back to a basal level, suggesting that the GIT of animals in certain conditions is eventually able to maintain and regulate its own immune system. 

#### 4.2.3. Implications

These heat maps suggest that a dose/exposure relationship exists, with high doses repressing intestinal cytokine expression over time, whereas low doses promote a rapid mucosal inflammatory response, and compromise Th1 and Treg responses over time. Importantly, the major point in our analysis concerns the ability of low doses to upregulate the intestinal expression of pro-inflammatory cytokines, especially following DON ingestion. This disturbance in cytokine balance could cause intestinal disorders. For instance, cytokines have a key function in the regulation of tight junction (TJ) proteins [96]. These proteins seal the space between two neighboring cells (see further explanations in the next section). However, upregulation of cytokines has been related to increased permeability that could allow the entry of luminal antigens and bacteria normally restricted to the GIT lumen. Translocation of bacteria has been already mentioned in this review, and the contribution of the effect on cytokine balance should be considered. Interestingly, in 2008 [53], and then in a review in 2010, Maresca and Fantini [97] provided evidence that several mycotoxins induce intestinal alterations that are similar to those observed at the onset and during the progression of inflammatory bowel diseases in human (among them upregulation of cytokines, increased permeability, and bacteria translocation). Although the life span of animals is relatively short in comparison to humans, the daily feeding of animals with material contaminated by mycotoxins could pose a serious risk of induction and persistence of chronic intestinal inflammation.

## 5. Consequence of Mycotoxins on Barrier Integrity

Intestinal epithelial cells (IECs) have two crucial but conflicting processes. On one hand, they transport nutrients and fluids and, on the other hand, they restrict the access for luminal antigens to the internal milieu. They form a monolayer that constitutes a dynamic and selective barrier, and mediates the transport of molecules in two ways: either across the cells (*i.e.*, the transcellular pathway) or between the cells (*i.e.*, the paracellular pathway) (Figure 2). This polarized monolayer effectively separates the apical (luminal) from the basolateral compartment, *i.e.*, the lamina propria. Tight junctions between adjacent cells represent an integral part of this compartmentalization and any damage to them leads to an enhanced permeability of the cell layer and a decreased transepithelial electrical resistance (TEER) which can lead to intestinal disorders. 

Since 2000, many authors have focused on the effect of mycotoxins on intestinal permeability. Modulation of the intestinal barrier was mostly studied with *in vitro* models (Table 5). Once differentiated and formed into a polarized monolayer, IECs become a very useful tool. Measurement of TEER is then feasible, and is considered as a good indicator of the integrity of epithelial barrier. At different concentrations, mycotoxins, particularly DON, are able to significantly reduce the TEER (Table 5). This decrease could indicate an alteration in paracellular permeability, but this ion movement across the monolayer can, however, be caused by changes in transcellular ion flux through altered plasma membrane channels or pumps. Accordingly, to eliminate this possibility, some authors have evaluated the apical to basolateral flux of paracellular markers, such as dextrans or mannitol, and noticed an increased flux after either DON or OTA exposure (Table 5) [30,54,55,56]. Effects in the paracellular pathway suggest an adverse effect on tight junctions (TJ). These are multiprotein complexes that link adjacent epithelial cells near their apical border (Figure 2). 

**Table 5:** Modulation of the intestinal barrier function by mycotoxins in *in vitro*, *ex vivo* and *in vivo* models.

		Teer	Paracellular Flux	Junction Proteins
**DON**	(RD)	*IPEC-1*: reduced TEER [54]. *IPEC-J2*: reduced TEER [50]. *Caco-2*: reduced TEER [54,55].	*Caco-2*: increased paracellular flux of mannitol [55].	*IPEC-J2*: reduced expression of ZO-1 [50,57] and claudin 3 [50]. *IPEC-1*: disappearance of ZO-1 [57]. *Caco-2*: reduced expression of claudin 4 but not occludin [55]. *Pig*: reduced expression of claudin 4 in jejunum [54], occludin & E-cadherin in ileum [51].
	(OD)	*IPEC-1*: reduced TEER [52,54,56].	*IPEC-1*: increased paracellular flux of 4-kDa dextran [54,56] and pathogenic *E.coli* [54]. *Pig explant*: increased paracellular flux of 4-kDa dextran [54].	*IPEC-1*: reduced expression of claudins 4 [52,54,56] & 3 but not ZO-1 and occludin [54].

**OTA**	(UD)	*Caco-2*: reduced TEER [29,30,31]. *HT-29*: reduced TEER [29].	*Caco-2*: increased in the paracellular flux of 4- and 10-kDa dextrans, but not 20- and 40-kDa dextrans [30].	*Caco-2*: disappearance of claudins 3 & 4 but not claudin 1 [30,31], ZO-1 and occludin [30].

**AF**	(RD)	*Caco-2*: slightly reduced TEER [25].		
	(UD)	*Caco-2*: reduced TEER [26].		

**FB**	(RD)			*Pig*: reduced expression of occludin & E-cadherin in ileum [51].
	(OD)	*IPEC-1*: reduced TEER [73].		

OD, Occasional Doses; RD, Realistic Doses; TEER, Transepithelial Electrical Resistance; UD, Unrealistic Doses.

Proteins present in the TJ complexes include ZO-1, occludin, and one or more claudin isoforms. TJ seal the luminal end of the intercellular space and limit transport by this paracellular route to relatively small hydrophilic molecules. ZO-1 acts as a scaffold to organize transmembrane TJ proteins and recruits various signaling molecules to the complex. Occludin binds to ZO-1 and the actin cytoskeleton and appears to have a role in regulating permeability through the TJ [30]. However, numerous studies have pointed to the claudin family of TJ proteins as a key determinant of paracellular characteristics. These proteins appear to form the backbone of the TJ. Therefore, it was of interest to explore the effect of these fungal metabolites on the TJ network, especially on claudins, and to relate these findings to the reduced TEER and the increased permeability to markers previously observed by several authors. Either by immunofluorescence or immunoblotting, it appears that DON and OTA removed or reduced the expression of claudin 4 and 3 on IECs (Table 5) [30,31,50,52,54,55,56]. Importantly, the finding on claudin 4 was demonstrated *in vivo* in the jejunum of pigs fed low concentrations of DON for five weeks [54]. Since DON exposure resulted in a reduction in total protein synthesis [55], the reduced expression of claudin 4 was attributed to this phenomenon rather than increased degradation or delocalization. Indeed, Van de Walle *et al.* [55] noted that claudin 4 expression was not restored after using an inhibitor of protein degradation, and Pinton *et al.* [54] did not observe delocalization of this protein from IECs plasma membrane. Further, both authors failed to show an effect at the mRNA level. In line with that, Lambert *et al.* [31] suggested that the delivery of *de novo* claudins 3 and 4 to the TJ complex might be perturbed by mycotoxins and would result in a reduction of total cellular levels of these claudins. As a consequence, new molecules would not arrive at the TJ to replace molecules which have been turned over. 

Oxidative stress could also participate in the effect of OTA on intestinal permeability [29,31]. Although FB_1_ has been shown to reduce the TEER in IECs [73], no data on the claudins is available. However, because of the well known effect of FB_1_ on sphingolipid metabolism [98], and the major role played by sphingolipids and lipid rafts in the establishment and maintenance of TJ [31], the gut epithelium might be prone to the adverse effects of FB_1_ on the barrier function. In accordance with this, Bracarense *et al.* [51] observed a defective expression of occludin and E-cadherin in the ileum of piglets fed low doses of FB_1_. In addition to the TJ complex, E-cadherins also play an important role in cell adhesion (Figure 2). With regard to occludin and ZO-1, inconsistent results have been reported (Table 5). Some authors did not report any effects of mycotoxins on the two proteins unlike claudins in their studies [30,54,55]. On the other hand, some authors showed a reduced expression of occludin or ZO-1 which was associated or not with an effect on claudins [50,51,57]. Interestingly, Diesing *et al.* [50] observed changes in TEER and in TJ proteins (ZO-1 and claudin 3) only when DON was applied on the basolateral side of IECs. The authors failed to show any effects of the mycotoxin after apical exposure, unlike other studies. This work provides a new insight into the effects of DON as there is evidence of the existence of an active DON transport in the basolateral to apical direction as opposed to simple diffusion from apical to basolateral in IECs [50]. Moreover, the application route of DON may explain why some authors failed to report effects on ZO-1 after apical incubation (Table 5). Similarly, change in occludin expression has been only reported *in vivo*, and not after *in vitro* apical exposure. 

Further work has been done to elucidate the initial cellular mechanism leading to this disruption of the intestinal barrier. TJ structure and function can be regulated by signaling molecules involved in MAPK pathways, and DON is known to rapidly activate MAPK [99]. Based on this observation, it has been demonstrated that DON decreases the intestinal barrier function through a MAPK dependent mechanism. Indeed, DON-activated MAPK led to a decrease in claudin expression [52,56], and inhibition of ERK1/2 phosporylation with a specific MAPK inhibitor restored the barrier function of IECs [56].

Collectively, these data suggest that some mycotoxins, especially DON have the ability to increase intestinal permeability, allowing the entry of luminal antigens normally restricted to the gut lumen. Translocation of bacteria across IEC monolayers have been already reported after DON exposure [53,54], and this event is considered as a key step in the induction and persistence of inflammatory bowel diseases [97]. Nonetheless, further studies with animals are required considering most of the data were from *in vitro* studies. Even if cellular models delineate the mechanism of action of the toxins, it is important to demonstrate that the same effect/mechanism can be observed on primary tissues. In this regard, Pinton and co-workers assessed the intestinal toxicity of mycotoxins *in vitro*, using IEC line, *ex vivo*, using intestinal explants and *in vivo*, using intestinal tissues from animals exposed to mycotoxin contaminated diets [52,54]. It can be concluded that mycotoxins could promote intestinal disorders, and coupled with the previous findings on Na^+^-dependent glucose transport, could be the underlying cause of diarrhea in animals exposed to mycotoxins. More importantly, mycotoxins may also facilitate their own intestinal absorption through the paracellular pathway. Some authors reported an increased permeability to compounds up to 10-kDa [30,54] whereas the molecular mass of mycotoxins is usually less than 1-kDa. Furthermore, mycotoxins that are poorly absorbed in the intestine, such as FB, would reach the systemic circulation easier if the intestinal barrier is compromised. In line with that, ingestion of co-contaminated feed with DON and FB resulted in more pronounced effects than the ingestion of the mono-contaminated feed with FB [51,100]. However, the authors did not measure the content of this mycotoxin in the plasma of animals fed the contaminated diets. Similarly, it has been demonstrated that 15-ADON, an acetylated derivative of DON commonly produced together with DON, induced a greater impairment of the barrier function (as measured through TEER, TJ, MAPK) than DON [52]. These findings emphasize the need to assess the effect of feed contaminated with more than one mycotoxin. 

## 6. Consequence of Mycotoxins on Intestinal Microflora

In every species, intestinal microflora are an important factor for animal health because there are close links between the host and its’ intestinal microflora especially through immune responses and via the metabolic products of microbial fermentation. Thus an impaired balance of the intestinal microbiome, such as in a dysbiosis condition, could have many adverse effects on the health of the host. However, investigating the microbial community shift is still a complex and imprecise activity, partly due to the low culturability of many bacterial species from the gastrointestinal tract (which can vary from 10% to 50%) and the inability of classical bacteriological counts to illustrate the changes in individual species abundance of the microbial community. Although culture-independent methods, such as high-throughput sequencing platforms have been developed to overcome culturing biases, data on the influence of toxins on the intestinal microflora is still limited. These issues account for the lack of information of mycotoxin effect on the microbiome, in contrast to the data on eukaryotic cells. Most of the data available regarding mycotoxin interactions with the animal’s microbiome, concern the role of intestinal microfora in mycotoxin detoxification [101].

Mycotoxins not only undergo microbial metabolism in the rumen or intestine, but may affect the microbes and their communities as some toxins exhibit antimicrobial properties [16]. Such antimicrobial activities of mycotoxins are suspected of (i) affecting the fermentative capacity of the rumen [16] or (ii) favoring a shift toward intestinal aerobic bacteria such as observed in inflammatory bowel diseases [97]. The tolerance of ruminants to some mycotoxins is attributed to toxin metabolism by rumen microorganisms, the detoxifying potential of the rumen may be influenced by diet composition. Indeed, the intake of a diet low in structural and high in rapidly degradable carbohydrates will decrease rumen pH, and an acid pH has been demonstrated to inhibit the complete transformation of DON to its metabolites [47]. Using different concentrate proportions in ruminant rations, realistic doses of DON decreased the fermentation of fiber fractions at the lower pH value [47,48] indicating a restriction of cellulolytic microbes. The change in the microbial community of the genus *Clostridium*, which contains cellulolytic species, after inclusion of DON confirmed these findings [47]. As reviewed by Maresca and Fantini [97], mycotoxins might be potential risk factors for chronic intestinal inflammatory diseases. In line with that, the total number and composition of intestinal microflora are significantly modified in inflammatory bowel diseases, with an increase in the number of aerobic bacteria and a parallel decrease in the number of anaerobic bacteria. Feeding pigs with T-2 toxin resulted in a substantial increase of aerobic bacterial counts in the intestine [65]. Similarly, chronic exposure of pigs to low doses of DON caused an increase in the number of intestinal aerobic bacteria and modified the dynamics of the intestinal bacteria communities [49]. By contrast, the mycotoxin FB_1_ did not alter the *in vitro* growth of isolated bacteria representative of intestinal microflora [72].

## 7. Conclusions

Mycotoxin research into effects on intestinal functions has made substantial progress in recent years By contrast to the limited distribution of mycotoxins into systemic tissues, the GIT is exposed to all the mycotoxins in contaminated feed. This suggests that the intestinal epithelium is the major site for the effects of mycotoxin contaminated material, even low levels of contamination. The influence of mycotoxins on intestinal balance is observed at relatively low levels that are not associated with obvious adverse effects on growth. Collectively, the data from research studies with realistic doses show that mycotoxins, and in particular DON, can compromise several intestinal functions, such as digestion, absorption, permeability, defense, and result in lower productivity and poor health of animals. In the future, much attention should be paid to low concentrations of mycotoxins, even though moderate doses can be encountered occasionally during unfavorable weather conditions. Besides, the consequences on physiological processes might be very different from those observed with high doses. Indeed, in this review we showed that low doses of mycotoxins are able to upregulate cytokine expression. By contrast, higher doses would lead to an opposite profile by downregulating them. Applegate *et al.*, [19] reported a similar effect on sialic acid excretion and the activity of intestinal maltase when using increasing concentrations of AF. The authors suggested that these physiological responses were following a pattern of hormesis. Hormesis is a dose-response phenomenon characterized by low-dose stimulation and high-dose inhibition. Hormesis has been noted in regards to changes in body weight of chickens receiving graded levels of dietary AF [102]. 

As shown in the Table 1, experiments elucidating the effects of mycotoxins other than DON on the GIT are rather limited. Similarly, more studies should investigate the effects of diets contaminated by more than one mycotoxin. Most fungi are able to produce several mycotoxins simultaneously and the mycotoxins produced depends on the feedstuff and crop growing conditions [3] and this has been demonstrated in worldwide mycotoxin surveys [6]. As it is a common practice to use multiple grain sources in animal diets, the risk of exposure to several mycotoxins increases with diet complexity [91]. Authors should experiment with naturally contaminated feed as feeding naturally contaminated grains take into account the presence of masked mycotoxins and their precursors [103,104] as well as unidentified fungal metabolites that may contribute to an underestimation of the total amount of mycotoxins. Sometimes, these factors can make interpretation of results difficult. Moreover, the nutritive value of grains may be lower due to fungi invasion, and thereby may cause a greater effect on animal productivity [3]. In a future where climate change may significantly affect the worldwide distribution and contamination by mycotoxigenic fungi and mycotoxins [105], the analysis of levels of contamination as well as the implementation of prevention and control strategies will be of major concern.
